# Endocrinological aging drives alterations in white matter architecture: Evidence from a humanized APOE risk model of Alzheimer’s disease

**DOI:** 10.1002/alz.092658

**Published:** 2025-01-09

**Authors:** Adam C. Raikes, Avnish Bhattrai, Tian Wang, Roberta Diaz Brinton

**Affiliations:** ^1^ University of Arizona, Tucson, AZ USA

## Abstract

**Background:**

Alzheimer’s affects women 2:1 compared to men, suggesting sex‐specific factors driving risk. Menopause, a female‐specific phenomenon, induces a multi‐system response across endocrine, metabolic, and immune‐inflammatory systems. Despite known effects on these systems, the impact on the brain and AD risk remains incompletely understood, limiting preventative options. Here, we investigated the impact of the menopausal transition on white matter properties in a late‐onset AD mouse model.

**Methods:**

6‐, 9‐ and 15‐month humanized APOEε3/ε3 (JAX #29018), ε4/ε4 (JAX #27894), and ε3/ε4 (bred in‐house) female mice were stratified into endocrine aging groups based on vaginal cytology profiles: regular (consistent 4–5 day cycles) or irregular (6–9 day cycles) cyclers or acyclic (no cycling >9 days). Ex‐vivo diffusion‐weighted imaging was used to analyze white matter fiber density (FD), cross‐section (FC), and their product (FDC), in addition to anisotropy (FA) and diffusivity (MD). Data were analyzed using generalized additive models with the main effects of endocrine aging (ref: 9‐month regular), hAPOE genotype, and total brain volume (except FD). Findings are reported at FDR corrected p<0.05.

**Results:**

Compared to 9‐month regular cyclers, both 6‐month regular and 15‐month acyclic mice had lower FC, primarily in bilateral fimbria, stria terminalis, corpus callosum, and gray matter regions, including bilateral hippocampal subfields and cingulate cortex (15‐month acyclic mice only). 15‐month acyclic mice showed lower FD/FDC in the left cingulate, piriform, and bilateral entorhinal cortices and greater FD/FDC in the bilateral habenular commissure and dorsal tenia tecta. FA and MD were significantly lower in the 15‐month acyclic mice in most white matter regions. Compared to 15‐month irregular cyclers, 15‐month acyclic mice exhibited lower FA in white matter tracts and striatal projection fibers.

**Conclusions:**

In these mice, menopause, rather than age or APOE genotype, altered white matter bundle properties, shifting toward smaller bundles of varying compactness and reduced bundle coherence. Pronounced differences were seen between 9‐month pre‐menopausal and 15‐month post‐menopausal mice. Differences between peri‐ and post‐menopausal mice at 15 months were only observed in FA. These findings implicate endocrine aging as a driving factor in white matter changes that may precede AD presentation. Ongoing work is focused on conducting translational analyses in the human brain.

